# Risk and protective factors for structural brain ageing in the eighth decade of life

**DOI:** 10.1007/s00429-017-1414-2

**Published:** 2017-04-19

**Authors:** Stuart J. Ritchie, Elliot M. Tucker-Drob, Simon R. Cox, David Alexander Dickie, Maria del C. Valdés Hernández, Janie Corley, Natalie A. Royle, Paul Redmond, Susana Muñoz Maniega, Alison Pattie, Benjamin S. Aribisala, Adele M. Taylor, Toni-Kim Clarke, Alan J. Gow, John M. Starr, Mark E. Bastin, Joanna M. Wardlaw, Ian J. Deary

**Affiliations:** 1 Department of Psychology, The University of Edinburgh, Edinburgh, UK; 2 Centre for Cognitive Ageing and Cognitive Epidemiology, The University of Edinburgh, Edinburgh, UK; 3 Department of Psychology, University of Texas, Austin, TX USA; 4 Brain Research Imaging Centre, The University of Edinburgh, Edinburgh, UK; 5 Scottish Imaging Network, A Platform for Scientific Excellence (SINAPSE) Collaboration, Edinburgh, UK; 6 Centre for Clinical Brain Sciences, The University of Edinburgh, Edinburgh, UK; 7 Computer Science Department, Faculty of Science, Lagos State University, Lagos, Nigeria; 8 Division of Psychiatry, The University of Edinburgh, Edinburgh, UK; 9 Department of Psychology, Heriot-Watt University, Edinburgh, UK; 10 Alzheimer Scotland Dementia Research Centre, The University of Edinburgh, Edinburgh, UK

**Keywords:** Ageing, Longitudinal, Structural MRI, Genetic, Lifestyle, Prediction

## Abstract

Individuals differ markedly in brain structure, and in how this structure degenerates during ageing. In a large sample of human participants (baseline *n* = 731 at age 73 years; follow-up *n* = 488 at age 76 years), we estimated the magnitude of mean change and variability in changes in MRI measures of brain macrostructure (grey matter, white matter, and white matter hyperintensity volumes) and microstructure (fractional anisotropy and mean diffusivity from diffusion tensor MRI). All indices showed significant average change with age, with considerable heterogeneity in those changes. We then tested eleven socioeconomic, physical, health, cognitive, allostatic (inflammatory and metabolic), and genetic variables for their value in predicting these differences in changes. Many of these variables were significantly correlated with baseline brain structure, but few could account for significant portions of the heterogeneity in subsequent brain change. Physical fitness was an exception, being correlated both with brain level and changes. The results suggest that only a subset of correlates of brain structure are also predictive of differences in brain ageing.

**Electronic supplementary material:**

The online version of this article (doi:10.1007/s00429-017-1414-2) contains supplementary material, which is available to authorized users.

## Introduction

Human brain structure shows several changes as people age. Healthy brain tissue atrophies in later life, with average losses of around 0.5% of total brain volume per year (Fotenos et al. 2005). Features such as white matter hyperintensities increase in size (Schmidt et al. 2005). Changes in white matter microstructure, measured using diffusion tensor (DT) MRI, also occur, with white matter tracts showing decreases in fractional anisotropy (FA; indicating the directional coherence of the movement of water molecules in the tracts; Kochunov et al. 2012) and increases in mean diffusivity (MD; indicating the average molecular motion in the tracts; Hsu et al. 2010; Muñoz Maniega et al. In press). Alterations in these key neural parameters have been linked to decreases in cognitive ability (Kloppenborg et al. 2014; Lövdén et al. 2014; Ritchie et al. 2015a, b), and thus may form part of the neural basis of ageing-related cognitive decline (Salthouse 2011). Since cognitive decline—even without dementia—is an increasing economic and social burden in Western countries (Brayne 2007), understanding the predictors of later-life brain changes is of growing importance. Understanding why some individuals’ brains age more healthily than others’—that is, investigating what some researchers have termed “brain maintenance” (Nyberg et al. 2012)—is a critical step toward designing treatments and interventions to ameliorate cognitive decline.

Previous cross-sectional studies have discovered correlations between differences in adult brain macro- and micro-structure and lifestyle factors such as smoking and alcohol consumption (Hudkins et al. 2012; Oscar-Berman and Marinković 2007), health conditions (Qiu 2014; Raz et al. 2003), physical fitness (Sexton et al. 2015), allostatic load (a measure of accumulated stress; Booth et al. 2015), and genetic factors such as the *APOE* e4 allele (Laukka et al. 2015) and genetic risk for schizophrenia (Staal et al. 2000). Although there is mixed evidence regarding this latter association (Liu et al. 2016; van der Auwera et al. 2015), there is reason to hypothesize that a higher load of schizophrenia-related genes may predispose individuals to poorer brain health, since strong genetic correlations have been found between schizophrenia and various cognitive abilities (Hagenaars et al. 2016).

Importantly, only some of these variables have been investigated as predictors of longitudinal changes in brain structure, and they have rarely been examined simultaneously. Poorer physical fitness (Tian et al. 2015), higher levels of glycated haemoglobin (Enzinger et al. 2005), and hypertension (Raz et al. 2003) have individually been associated with accelerated rates of brain atrophy. There is conflicting evidence for the role of the *APOE* e4 allele in brain tissue loss in individuals without dementia (Josephs et al. 2008; Wishart et al. 2006). Risk factors for the accumulation of white matter hyperintensities, which have been studied in more depth than changes in healthy brain tissues, tend to be vascular-related (Wardlaw et al. 2015). Very few longitudinal studies have examined predictors of later-life change in white matter diffusion parameters (see Köhncke et al. 2016, for the one example of which we are aware). The distinction between cross-sectional and longitudinal analyses of brain ageing is important: in cross-sectional studies, it is not possible to differentiate between developmental processes that occur in earlier periods of life from effects that are specifically ageing-related (Tucker-Drob and Salthouse 2011).

The present study reports an analysis of predictors of change in brain volumes (grey matter, normal-appearing white matter, and white matter hyperintensities) and diffusion parameters (latent general factors of fractional anisotropy and of mean diffusivity, extracted from diffusion tensor imaging of multiple white matter tracts across the brain; see Methods) between age 73 and 76 years. We selected a wide range of potential predictors on the basis of the literature surveyed above, examining health, fitness, lifestyle, cognitive, socioeconomic, and genetic variables. In order to reduce the numerous predictor variables to a manageable number, where possible we extracted latent variables that indexed shared variance between groups of predictors. The final predictor list was the following: sex, physical fitness, allostatic load, health conditions, socioeconomic status, prior intelligence, education, smoking, alcohol consumption, *APOE* e4 status, and polygenic risk for schizophrenia. We tested the predictors’ relation to brain changes. First, we examined the value of each predictor individually. Second, we used penalized regression (Zou and Hastie 2005) to create parsimonious multivariate models that predicted each brain outcome from an optimal combination of predictors.

## Materials and methods

### Participants

The Lothian Birth Cohort 1936 (Deary et al. 2007, 2012) is a longitudinal study of ageing based in the Edinburgh and Lothians area of Scotland, United Kingdom. Participants were invited to join the study on the basis of their participation, at age 11, in the Scottish Mental Survey of 1947, during which most had completed a test of general intelligence (see below). They were followed up in later life and have so far completed three waves of later-life testing. At the first wave, in 2004–2007, participants were aged 69.53 years on average (SD = 0.83; *n* = 1,091; 543 females). At the second wave, in 2007–2010, they were aged 72.49 years (SD = 0.71; *n* = 866; 418 females). At the third wave, in 2011–2014, they were aged 76.25 years (SD = 0.68, *n* = 697; 337 females). The majority of the ‘predictor’ variables in the present study, described below, were measured at the second testing wave.

At the second and third waves, the participants were also invited to attend for a structural and diffusion tensor brain scan (Wardlaw et al. 2011). 731 participants (343 females) were scanned at the second wave (mean age: 72.68 years; SD = 0.72), and 488 (228 females) were scanned at the third wave (mean age: 76.38 years; SD = 0.65). Different amounts of usable data were obtained for each of the brain measures: the valid sample sizes for each measure are shown in Table 2.

The study was approved by the Multi-Centre Research Ethics Committee for Scotland (MREC/01/0/56) and the Lothian Research Ethics Committee (LREC/2003/2/29). All participants completed written informed consent forms before any cognitive, MRI, or other measurements were taken.

### Measures

#### MRI scanning

All participants were scanned using the same scanner (1.5 T GE Signa Horizon HDx; General Electric, Milwaukee, WI, USA), using the same protocol, at both waves. The full imaging protocol is described elsewhere (Wardlaw et al. 2011).

#### Brain volumetric measures

**Fig. 1:**
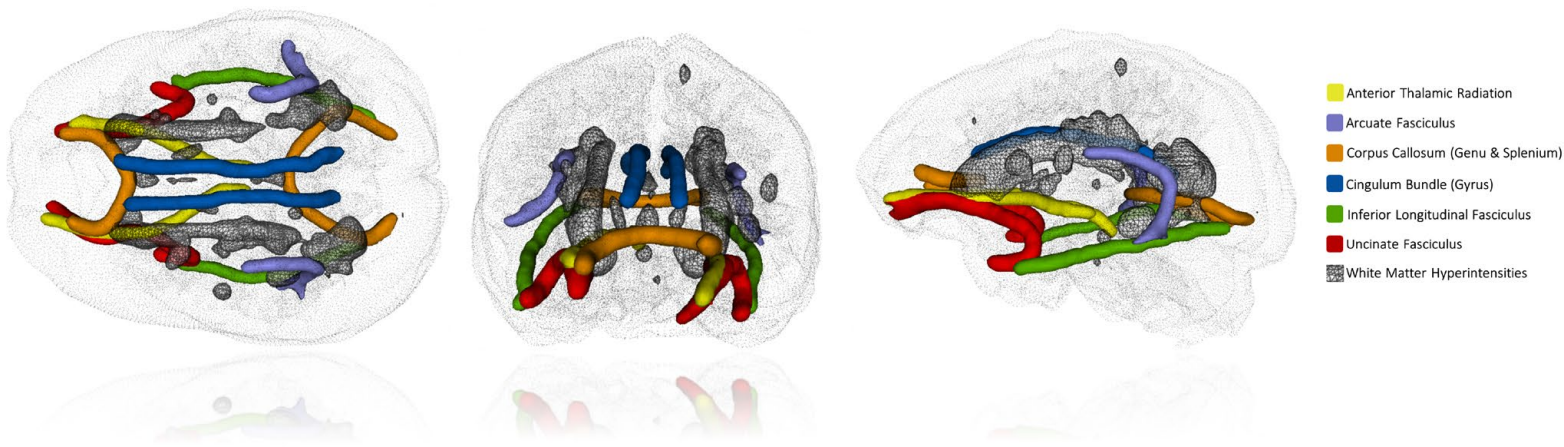
Illustration of the 12 white matter tracts (five bilateral; two from the corpus callosum) measured using probabilistic neighborhood tractography in one Lothian Birth Cohort 1936 study participant (in color). Also shown are the volumetrically-estimated white matter hyperintensities observed in this participant (crosshatched). Tracts and hyperintensities are displayed inside the participant’s spatially-registered T1-weighted brain volume

We measured brain volumes (specifically grey matter, normal-appearing white matter, and white matter hyperintensity volumes) using a validated multispectral image processing method that combines T1-, T2-, T2^*^-, and FLAIR-weighted MRI sequences for segmentation (based on a previous method; Valdés Hernández et al. 2010). According to STandards for ReportIng Vascular changes on nEuroimaging (STRIVE), we explicitly defined white matter hyperintensities as punctate, focal, or diffuse lesions in all subcortical regions (Wardlaw et al. 2013). We manually checked all segmented images for accuracy blinded to all participant characteristics, corrected errors, and excluded imaging-detected infarcts from white matter hyperintensity volumes (Wang et al. 2012). White matter hyperintensity volume was log-transformed before inclusion in the analyses, since it had a right–skewed distribution (that is, many participants had few hyperintensities). White matter hyperintensities are illustrated as the crosshatched areas in Fig. 1, alongside the diffusion-tensor measures described below. Note that the analyses below are performed on the total tissue volumes; when adjusting these volumes for intracranial volume, the pattern of results—especially for longitudinal changes (the main focus of the report)—was nearly identical.

### White matter diffusion-tensor measures

All diffusion MRI data were converted from DICOM (http://dicom.nema.org/) to NIfTI-1 (http://nifti.nimh.nih.gov/nifti-1/) format using TractoR tools (http://www.tractor-mri.org.uk; Clayden et al. 2011), and pre-processed with FSL tools (http://www.fmrib.ox.ac.uk/fsl/; Jenkinson et al. 2012) in order to extract the brain, eliminate bulk patient motion and eddy current-induced artifacts, and estimate FA and MD in each brain voxel. The underlying connectivity data was generated using BedpostX/ProbTrackX (Behrens et al. 2007), with a two-fiber model and 5000 streamlines to reconstruct tracts of interest.

As described in detail previously (Ritchie et al. 2015a), 12 tracts of interest were identified in each participant using probabilistic neighborhood tractography, an automatic tract segmentation method with good reproducibility (Clayden et al. 2009). The 12 tracts segmented were the genu and splenium of corpus callosum; the bilateral cingulum cingulate gyri; the arcuate, uncinate, and inferior longitudinal fasciculi; and the anterior thalamic radiations. Tract-averaged values of MD and FA, weighted by connection probability, were generated for each pathway in those tracts that were visually assessed to be anatomically plausible representations of the fasciculus of interest. The tracts are illustrated in color in Fig. 1.

At each scanning wave, we estimated latent general factors of FA and MD from tract-averaged estimates from each of the measured tracts. These general factors capture the shared variance in FA or MD across multiple tracts. Similar analyses have been used in several previous studies of white matter microstructure (Ritchie et al. 2015a; Bender et al. 2016). All latent variable loadings were highly significant and mostly moderate-to-large in size, except for the loading of MD in the splenium of the corpus callosum on general MD at each wave, which were weak (0.200 at age 73 and 0.275 at age 76). For this reason, we did not use this tract as part of the general MD factor, reducing the number of indicators for MD at each wave to 11. All 12 tracts were used to indicate the general FA factor at both waves.

#### Predictors of brain change

We selected 11 candidate predictors of brain change. For four of the predictors, we created latent variables from multiple manifest indicators. Physical fitness comprised grip strength in the dominant hand (measured on a Hydraulic Hand Dynamometer), forced expiratory volume in 1s (measured using a Micro Medical Spriometer), and 6-metre walk time, all measured at age 73. All three physical variables were adjusted for height. Allostatic load, a variable theoretically representing ‘wear and tear’ on bodily systems (McEwen 1998), was indicated by a variety of inflammatory biomarkers (fibrinogen, C-reactive protein, interleukin-6) and metabolic biomarkers (triglycerides, glycated haemoglobin, low- and high-density lipoprotein, and body mass index), all derived from blood samples or direct measurements taken at age 73 (Booth et al. 2015). Socioeconomic status was indicated by the participant’s father’s occupational class at their birth in 1936, the participant’s own highest achieved occupational class before retirement, and the Scottish Index of Multiple Deprivation, a government-collected, neighborhood-level indicator of social deprivation, estimated for the present participants at age 70 (Executive 2006). Prior intelligence was indicated by the participant’s scores on three tests: the Moray House Test No. 12 (Scottish Council for Research in Education 1949), taken at age 11, the National Adult Reading Test (Nelson and Willison 1991), and the Wechsler Test of Adult Reading (Wechsler 2001). The latter two tests were taken at age 70, during the first wave of the study.

The remaining seven predictors were included in the model as manifest (i.e. single) variables. Sex was indicated at entry to the study. Health conditions were measured as a sum of the number of the following conditions the participants reported suffering from: diabetes, hypertension, stroke, and cardiovascular disease (since these were all binary variables, we chose to calculate a sum rather than a latent variable). Education was self-reported by the participants as the number of years of formal, full-time education the participants completed. Smoking status was recorded as current, ex-, or never, reported at age 73. Alcohol consumption was recorded as grams per week, as reported on a validated Food Frequency Questionnaire at age 70 (this variable was log-transformed to normalize its distribution). *Apolipoprotein (APOE) e4 genotype* was derived from blood samples using TaqMan technology at the Wellcome Trust Clinical Research Facility Genetics Core at the Western General Hospital, Edinburgh. The *APOE* variable used here classifies participants into ‘no e4 alleles’ versus ‘one or two e4 alleles’. Finally, each participant’s regression-weighted polygenic risk score for schizophrenia was derived from single-nucleotide polymorphism (SNP) genotyping, using summary data from the most recent genome-wide association study for schizophrenia (Schizophrenia Working Group of the Psychiatric Genomics Consortium 2014; see previous work in this sample by McIntosh et al. 2013, for more details about risk score calculation). For the present study, we used the most liberal SNP inclusion threshold (all SNPs, *p* = 1.00). When using the polygenic score, we also included four multidimensional scaling components to control for population stratification. Participants also completed the Mini-Mental State Examination (MMSE; Folstein et al. 1975) at both waves. This was used as part of a secondary analysis, described below.

### Statistical analyses

We estimated three structural equation models, each including different brain measurements as the dependent variables. These were change score models (McArdle 2009), which allow the assessment of levels (in this case, the brain’s baseline status at age 73) and changes across two measurement waves (brain changes between age 73 and age 76).

**Fig. 2:**
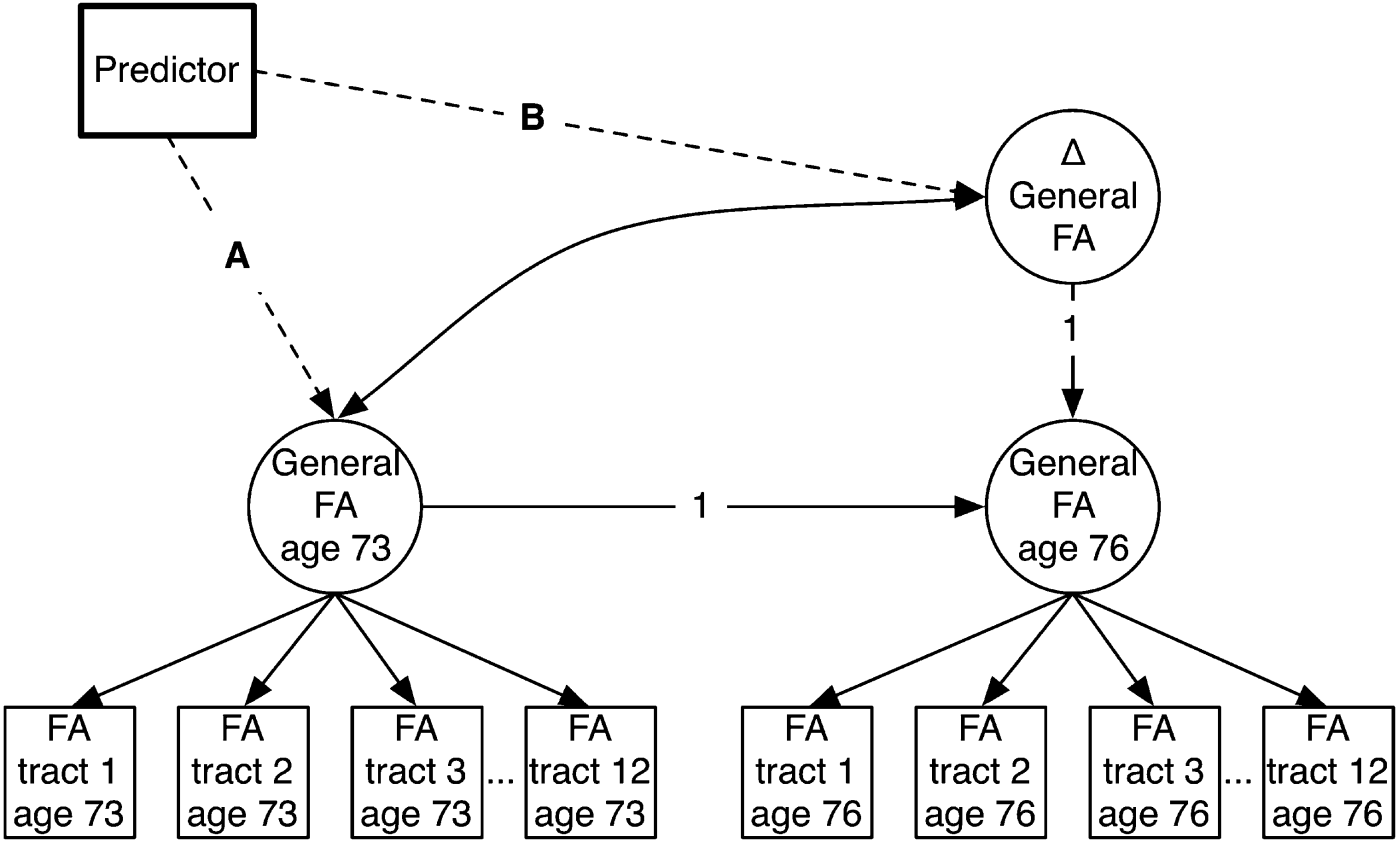
Simplified diagram of the structural equation model for the general fractional anisotropy (*FA*) variable. FA in white matter tracts 1–12 was measured at baseline (age 73) and follow-up (age 76), and a General FA factor extracted at each wave. From these a latent change score variable (Δ General FA) was calculated. Then, each of the predictor variables was assessed for its relation with baseline level (*path A*) and change (*path B*). The equivalent model for general mean diffusivity used only 11 tracts. The equivalent models for *grey matter* volume, *white matter* volume, and *white matter* *hyperintensity* volume did not use latent factors at each wave

Model 1 had as its dependent variables three brain volumetric changes: changes in the volumes of grey matter, normal-appearing white matter, and white matter hyperintensities. Model 2 used the FA values from the DT-MRI scans, producing a latent variable of general FA across the 12 tracts at each scanning wave, and a latent change score variable (ΔFA) indexing the FA change across the waves. Model 3 used the MD values from the same DT-MRI scans, producing a latent variable of cross-wave MD change (ΔMD). Figure 2 shows a simplified diagram of the model. For general FA and MD, we imposed strong measurement invariance across the waves (that is, we constrained the loadings and intercepts of each tract to equality across wave, under the assumption that the same latent trait was being measured across waves; see Widaman et al. 2010; Ritchie et al. 2015a) Since there was a small amount of within-wave age variability, models adjusted the brain variables for the participant’s age in days at the time of scanning. For each of the three models, we tested the association of brain baseline levels and changes with each individual predictor variable (some of which were latent variables, and some of which were manifest, as described above), by regressing the brain outcome on each predictor separately. All of these analyses also adjusted for sex. Structural equation modeling was performed in Mplus v7.3 (Muthén and Muthén 1998–2014), and used full-information maximum likelihood estimation in order to use all of the available data.

Next, we produced simultaneous, multivariate regression models that included a combination of the most appropriate predictor variables for each brain outcome. Methods of producing such models include forwards and backwards stepwise regression, but these can lead to overfitting, and thus a lower likelihood of findings that are replicable in independent datasets. For this reason, we used a penalized regression method, the elastic net (Zou and Hastie 2005), which combines the advantages of least absolute shrinkage and selection operator (LASSO) regression (production of a parsimonious model by removing irrelevant variables) and ridge regression (good performance in the presence of collinear predictors, as was the case in our dataset; Morozova et al. 2015). These analyses were performed using the ‘glmnet’ package for R (Friedman et al. 2010), with 10-fold cross-validation. We bootstrapped each model 1000 times, including in the final model only those variables that had a non-zero coefficient in 50% or more of the iterations. Thus, we selected variables that were stably chosen by the estimator as relevant predictors of each neuroanatomical outcome. We took these selected predictors and simultaneously entered them into the latent difference score models to produce the results described below.

## Results

Full descriptive statistics and valid sample sizes for all measures, along with the loadings of each variable on its factor (where applicable) are presented in Tables 1 (for predictors) and 2 (for brain parameters). Correlations among the predictor variables are shown in Table 3.

**Table 1:** Descriptive statistics and factor loadings for predictor variables

Variable category	Measured variable	*n*	Mean (SD) or *n* for categorical measures	Factor loading
Physical fitness (latent)	Grip strength	823	28.54 (9.39)	0.393
	Forced expiratory volume	856	2.30 (0.68)	0.458
	6 m walk time	860	4.35 (1.32)	0.482
Allostatic load (latent)	Fibrinogen	819	3.25 (0.61)	0.482
	C-reactive protein	830	4.94 (7.84)	0.506
	Interleukin-6	815	2.05 (1.73)	0.585
	Triglycerides	832	1.65 (0.82)	0.314
	Glycated haemoglobin	826	5.75 (0.66)	0.376
	Low-density lipoprotein	829	2.93 (1.04)	0.251
	High-density lipoprotein	832	1.46 (0.44)	0.341
	Body mass index	866	27.92 (4.45)	0.402
Socioeconomic status (latent)	Father’s occupational class (1–5)*	960	2.91 (0.94)	0.372
	Own occupational class (1–5)*	1091	3.54 (1.20)	0.505
	Scottish Index of Multiple Deprivation (1–8)*	1083	6.25 (2.09)	0.540
Prior intelligence (latent)	Moray House Test (age 11)	1028	100 (15.00)	0.731
	NART (max. 50)	864	34.38 (8.18)	0.958
	WTAR (max. 50)	864	41.01 (6.97)	0.944
Manifest (single) predictors	Health conditions (0–4)	854	0.95 (0.91)	–
	Education (years)*	1091	10.74 (1.13)	–
	Smoking	866	415 never; 378 ex; 73 current	–
	Alcohol (g per week)*	928	11.98 (16.79)	–
	*APOE* e4	1028	306 with 1 or 2 e4 alleles; 722 with no e4 alleles	–
	Polygenic risk for SCZ	953	0.49 (0.02)	–
Dementia screening	MMSE age 73 (max. 30)	865	28.75 (1.42)	–
	MMSE age 76 (max. 30)	697	28.65 (1.70)	–

*Variables collected at age 70; all other variables collected at age 73, unless otherwise noted

**Table 2:** Descriptive statistics and factor loadings for brain measurements

Measured variable	Wave 2 (age 73)			Wave 3 (age 76)	
	*n*	M (SD)	Factor loading	*n*	M (SD)
Grey matter volume (cm^3^)	657	472.43 (44.68)	–	461	465.67 (43.61)
White matter volume (cm^3^)	657	476.89 (50.55)	–	461	464.25 (53.09)
White matter hyperintensity volume (cm^3^)	656	12.23 (12.18)	–	464	15.85 (14.57)
Corpus callosum genu FA	646	0.41 (0.05)	0.602	438	0.38 (0.04)
Corpus callosum splenium FA	663	0.49 (0.07)	0.338	437	0.51 (0.07)
L arcuate fasciculus FA	639	0.45 (0.04)	0.648	455	0.43 (0.04)
R arcuate fasciculus FA	580	0.43 (0.04)	0.613	414	0.41 (0.04)
L anterior thalamic radiation FA	556	0.32 (0.03)	0.642	429	0.33 (0.03)
R anterior thalamic radiation FA	643	0.33 (0.03)	0.657	453	0.34 (0.03)
L rostral cingulum FA	641	0.44 (0.05)	0.596	448	0.44 (0.05)
R rostral cingulum FA	650	0.39 (0.04)	0.551	448	0.41 (0.05)
L uncinate fasciculus FA	567	0.33 (0.03)	0.680	407	0.34 (0.03)
R uncinate fasciculus FA	628	0.33 (0.03)	0.670	443	0.33 (0.03)
L inferior longitudinal fasciculus FA	663	0.40 (0.05)	0.510	455	0.39 (0.06)
R inferior longitudinal fasciculus FA	664	0.38 (0.05)	0.480	462	0.38 (0.05)
Corpus callosum genu MD	646	770.05 (66.05)	0.659	438	850.28 (82.99)
Corpus callosum splenium MD	663	978.47 (173.32)	-	437	852.01 (153.16)
L arcuate fasciculus MD	639	659.50 (47.60)	0.686	455	699.45 (58.14)
R arcuate fasciculus MD	580	646.35 (52.29)	0.715	414	681.46 (58.21)
L anterior thalamic radiation MD	556	758.24 (66.00)	0.653	429	795.90 (66.85)
R anterior thalamic radiation MD	643	754.29 (62.44)	0.738	453	791.45 (86.75)
L rostral cingulum MD	641	648.13 (45.64)	0.557	448	673.84 (46.71)
R rostral cingulum MD	650	651.92 (45.08)	0.618	448	663.39 (42.12)
L uncinate fasciculus MD	567	770.37 (53.82)	0.643	407	794.30 (57.22)
R uncinate fasciculus MD	628	756.31 (52.30)	0.703	443	796.54 (58.11)
L inferior longitudinal fasciculus MD	663	771.85 (100.10)	0.426	455	826.54 (143.47)
R inferior longitudinal fasciculus MD	664	772.20 (101.48)	0.414	462	801.15 (122.90)

Factor loadings were invariant across waves. The splenium had a low loading for MD and so was not included in the general factor

*FA* fractional anisotropy; *MD* mean diffusivity; *L*/*R* left/right hemisphere

**Table 3:** Associations (standardized betas) among latent and manifest predictor variables, estimated within a structural equation model

Variable	1.	2.	3.	4.	5.	6.	7.	8.	9.	10.
1. Sex (male)	–									
2. Physical fitness^†^	0.01	–								
3. Allostatic load^†^	−0.02	−0.68^***^	–							
4. Health conditions	0.11^**^	−0.36^***^	0.37^***^	–						
5. Socioeconomic status^†^	0.05	0.38^***^	−0.35^***^	−0.11^*^	–					
6. Prior intelligence^†^	−0.09^**^	0.26^***^	−0.20^***^	−0.09^**^	0.69^***^	–				
7. Education	0.01	0.16^**^	−0.17^***^	−0.08^*^	0.71^***^	0.55^***^	–			
8. Smoking	−0.08^*^	−0.27^***^	0.25^***^	0.05	−0.21^***^	−0.07^*^	−0.11^**^	–		
9. Alcohol consumption	0.35^***^	0.12^*^	−0.18^***^	−0.05	0.35^***^	0.19^***^	0.19^***^	0.03	–	
10. *APOE* e4	0.04	0.08	−0.07	−0.03	0.04	0.03	0.002	−0.06	0.03	–
11. Polygenic risk for schizophrenia	0.04	−0.03	−0.02	−0.04	−0.003	−0.07	−0.02	0.06	0.06	−0.01

*p < 0.05, ** = *p* < 0.01; *** = *p* < 0.001. ^†^ = latent variable predictor

**Fig. 3:**
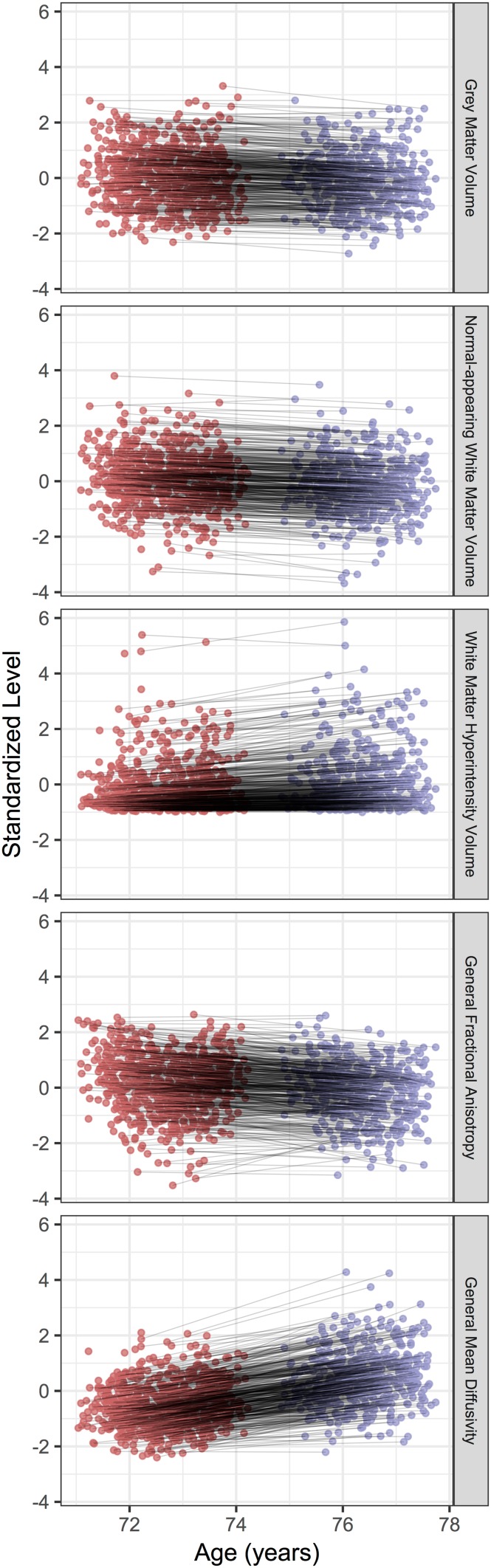
Change in each brain measure between age 73 and age 76 years, showing an individual point for each participant at the first scanning wave (*left, red cluster*) and the second (*right, purple cluster*). Participants who returned for the second scan have their points connected with a *grey line*. For comparison, all measurements are shown on a standardized scale

All brain measures showed statistically significant mean change across the 3 years of the study (all *p* values <0.001). The models implied that grey matter volume change was −0.07 SDs/year (−0.64% change/year). Normal-appearing white matter volume change was −0.10 SDs/year (−1.01%/year). White matter hyperintensity volume change was +0.11 SDs/year (+11.04%/year). General FA change was −0.09 SDs/year. General MD change was +0.34 SDs/year. Each of the brain changes is illustrated in Fig. 3; although mean change was observed, there was substantial between-person variation in the magnitude of these changes.

The base models for general FA and MD had adequate fit to the data (general FA: *χ* ^2^(251) = 639.44, *p* < 0.001, root mean square error of approximation (RMSEA) = 0.047, Comparative Fit Index (CFI) = 0.914, Tucker-Lewis Index (TLI) = 0.906; general MD: *χ* ^2^(207) = 618.86, *p* < 0.001, RMSEA = 0.059, CFI = 0.917, TLI = 0.907). Note that the base model for the three brain volumetric variables was saturated and thus had perfect fit to the data by definition.

### Individual predictors of brain structure baseline level and change

**Fig. 4:**
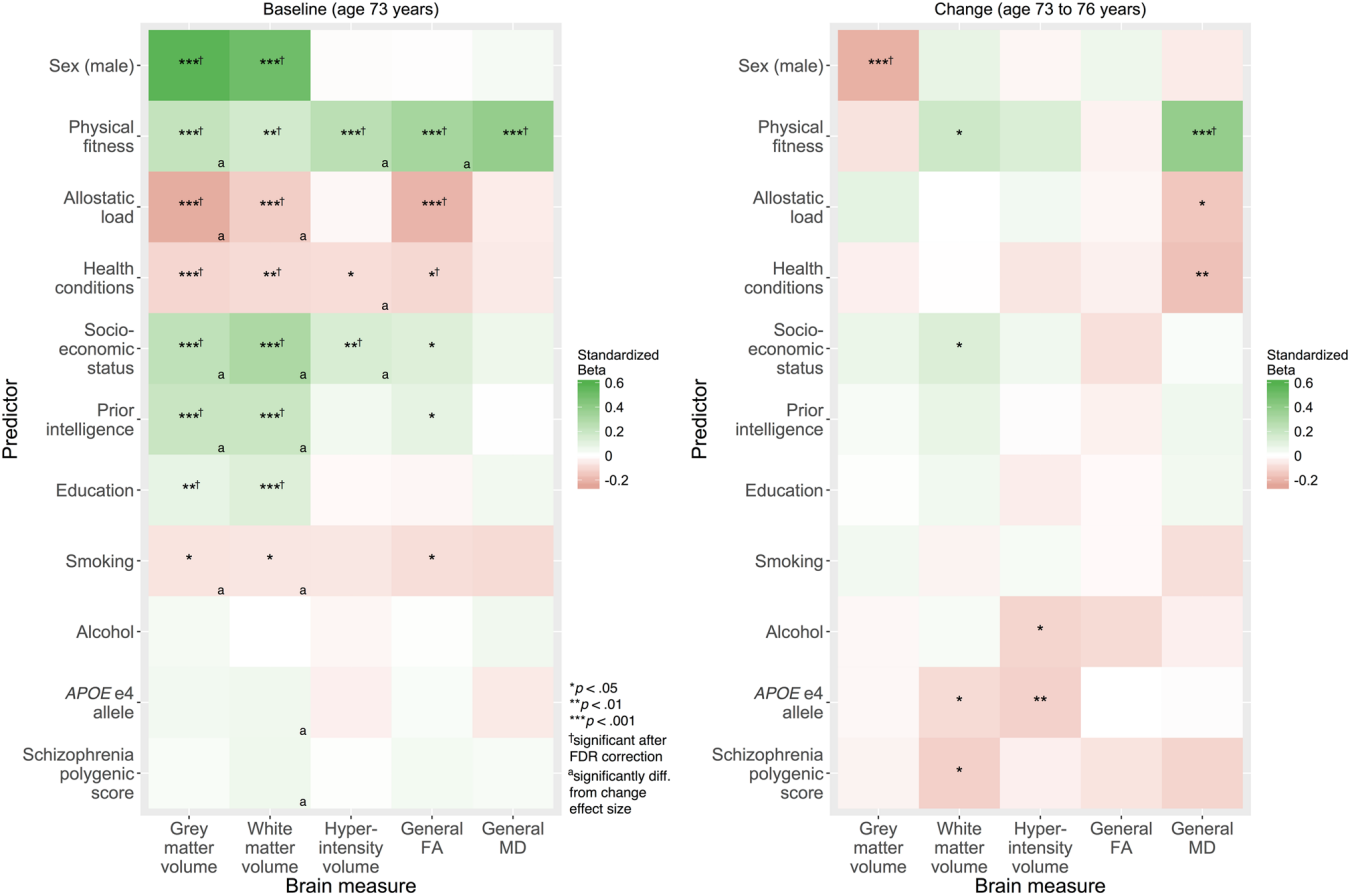
Associations of each predictor with baseline levels of (*left*) and changes in (*right*) each brain measure (all associations adjusted for age and sex). Brain variables scaled such that positive associations (*green*) indicate healthier brain baseline levels and brain ageing, and negative associations (*red*) indicate unhealthier brain baseline levels and brain ageing. Hyperintensity = white matter hyperintensity; *FA* fractional anisotropy; *MD* mean diffusivity. ^*^ *p* < 0.05, ^**^ *p* < 0.01, ^***^ *p* < 0.001; ^†^significant after false discovery rate (FDR) correction; ^a^effect size for baseline was significantly different from effect size for change

We regressed each brain baseline level and change variable on each of the predictors separately (also controlling for sex in each case; the effect of sex alone was also examined). The results of these analyses are shown in Fig. 4 (all values are provided in Supplementary Tables S1 for baseline level and Supplementary Table S2 for change). All results are reported with the brain outcomes scaled such that higher scores indicate healthier baseline levels (more grey and white matter, higher general FA, lower hyperintensity volume and lower general MD) and healthier changes (less decline in grey matter, white matter, and general FA; shallower increases in hyperintensity volume and general MD). That is, positive associations suggest healthier ageing and negative correlations suggest less healthy ageing.

Many associations were found between the predictors and brain baseline level in all five measures. The only variables that showed no significant relation with any baseline brain variable were alcohol consumption, *APOE* e4 status, and polygenic schizophrenia risk. We note that many of the predictor associations with brain baseline levels have been reported individually in previous publications on the Lothian Birth Cohort 1936 (Booth et al. 2015; Gow et al. 2012; Ritchie et al. 2015a, b); these results are provided here so as to evaluate multiple predictors together, and as a comparison to the new results for brain changes.

For change, there were far fewer associations. Only sex was associated with change in grey matter volume, with males declining faster. There were no significant relations with change in general FA. Some predictors had value, as shown in Fig. 4: physical fitness was significantly associated with healthier ageing in white matter volume and general MD, the *APOE* e4 allele was linked to less healthy changes in white matter and white matter hyperintensity volume, among some other, borderline associations. Importantly however, these results are uncorrected for multiple comparisons. Introducing a per-outcome false discovery rate correction (Benjamini and Hochberg 1995) reduced all change associations to non-significance except the association of sex with grey matter change, and the association of physical fitness with change in general MD (see dagger signs in Fig. 4).

We next explored the degree of correspondence between predictors of level and predictors of change. We did this in two ways. First, for those predictors that were significant (uncorrected) for either level or change, we tested whether the effect sizes for level and for change were significantly different from one another. As shown by the ‘a’ superscript in Fig. 4, we found that many of the associations that were significant for level were significantly different from the corresponding association for change (for example, the associations of SES, prior intelligence, and education with the volumetric measures). Some of the significant associations with change (namely the *APOE* e4 allele and the schizophrenia polygenic profile score) were significantly larger than those for level. Second, we tested the correlation of the vector of effect sizes for baseline level with the vector of effect sizes for change. Across all effect sizes for all brain variables, there was only a modest, non-significant correlation between the effect sizes (*r*(53) = 0.27, *p* = 0.05), suggesting relatively poor correspondence between the effect sizes. Overall, this indicated that obtaining a significant effect for a correlate of levels of late life brain structure cannot be regarded as prima facie evidence that the variable is a similarly-effective predictor of ageing-related structural change.

### Models with multiple predictors of baseline brain structure and change

The elastic net regression procedure selected variables to include in the simultaneous-predictor models, keeping each model as parsimonious as possible (Table 4). As shown previously (Morozova et al. 2015), penalized regression procedures often select variables that are not incrementally significant in a regression model, but that nonetheless reduce the mean squared error if they are included. This was the case for our models: the selection procedure selected several variables that were not significant in the model (the unbolded coefficients in Table 4). With only a few differences, the change results from the individual analyses remained intact in these simultaneous models: physical fitness and *APOE* e4 status remained the most substantial predictors of brain change, although some factors that were not previously statistically significant, such as alcohol consumption (in this case associated with white matter hyperintensity progression) became significant in the simultaneous model.

**Table 4:** Results from the simultaneous models, after variable selection by elastic net

Outcome type	Predictor	Association with brain outcomes														
		Grey matter volume			Normal-appearing white matter volume			White matter hyperintensity volume			General fractional anisotropy			General mean diffusivity		
		*β*	SE	*p*	*β*	SE	*p*	*β*	SE	*p*	*β*	SE	*p*	*β*	SE	*p*
Brain baseline level	Sex (male)	**0.609**	**0.026**	**<0.001†**	**0.576**	**0.033**	**<0.001†**							0.078	0.047	0.098
	Physical fitness	0.042	0.056	0.457	0.019	0.066	0.768	**0.144**	**0.066**	**0.029** ^†^	**0.180**	**0.069**	**0.009** ^†^	**0.181**	**0.074**	**0.015** ^†^
	Allostatic load	**−0.174**	**0.044**	**<0.001** ^†^	**−0.101**	**0.048**	**0.035**				−0.095	0.053	0.072			
	Health conditions	−0.059	0.035	0.094	−0.029	0.040	0.466				−**0.100**	**0.042**	**0.017** ^†^			
	SES	0.054	0.055	0.330	0.173	0.059	0.004†				0.047	0.068	0.489			
	Prior intelligence	**0.164**	**0.037**	<**0.001** ^†^	**0.116**	**0.047**	**0.013** ^†^				0.094	0.052	0.069			
	Education				0.002	0.046	0.966				− **0.138**	**0.050**	**0.006** ^†^			
	Alcohol consump				− 0.065	0.041	0.117									
	*APOE* e4 genotype	0.059	0.032	0.066	**0.074**	**0.037**	**0.043**									
	Polygenic SCZ risk	0.022	0.032	0.505	0.068	0.037	0.067									
Brain change	Sex (male)	−**0.199**	**0.049**	<**0.001** ^†^	0.090	0.052	0.084									
	Physical fitness				**0.259**	**0.096**	**0.007** ^†^	−0.065	0.077	0.396				**0.346**	**0.103**	**0.001** ^†^
	Allostatic load	0.103	0.059	0.083	0.107	0.063	0.091									
	Health conditions				−0.009	0.055	0.870							−0.114	0.059	0.055
	SES				0.132	0.072	0.068									
	Education							0.032	0.037	0.389						
	Smoking															
	Alcohol consump							−0.108	0.038	0.004†						
	*APOE* e4 genotype				−**0.152**	**0.050**	**0.002** ^†^	−**0.080**	**0.035**	**0.023**						
	Polygenic SCZ risk				−0.099	0.052	0.055	−0.039	0.037	0.290				−**0.120**	**0.059**	**0.041**

Blank cells indicate variables that were not selected for the relevant brain outcome. Bold values are statistically significant. All brain outcomes scored such that positive associations indicate links to healthier ageing, and negative associations indicate links to unhealthy ageing

Variables not selected for inclusion in the models for any brain outcome were smoking (for brain baseline levels) and intelligence (for brain changes). *p*-values shown uncorrected; those values that survived per-outcome False Discovery Rate correction are indicated with a ^†^ symbol

*SCZ* schizophrenia; *SES* socioeconomic status; *β* standardized beta

### Models excluding individuals with possible dementia

Finally, we re-ran our individual-predictor-variable analyses removing sixteen individuals with mini-mental state examination (Folstein et al. 1975) scores below 24 (a commonly-used cut-off that might indicate mild cognitive impairment or dementia) at either testing wave. Whereas the vast majority of the results were very similar to those from the main analysis, there were no longer any significant baseline level associations for smoking, or any significant change associations for possession of the *APOE* e4 allele.

## Discussion

This investigation of neurostructural changes between age 73 and age 76 makes two key contributions. First, it identifies some potentially important predictors of brain change. For example, physical fitness and *APOE* e4 were the most consistent predictors of differential rates of brain ageing, and are therefore promising foci for further investigation into predictions of brain change. On the whole, effect sizes for predictors of longitudinal brain change were small (all standardized *β* values < 0.4 for brain changes), and few were statistically significant. Predictors differed across the different neural outcomes under study, suggesting that ageing-related brain change is a multifaceted, multidetermined process (see e.g. Kievit et al. 2014). Second, the results highlight the important point that variables that are significantly associated cross-sectionally with baseline levels of neural structure do not necessarily make significant predictions about longitudinal neural changes. We found that many of the correlates of initial baseline levels of brain structure did not significantly predict subsequent rates of change in brain structure; in some cases predictor associations with change were in opposite directions from associations with baseline levels.

The fact that significant predictors of longitudinal brain changes were not always correlates of baseline levels of brain structure suggests that variables we found to be related to brain structure longitudinally but not cross-sectionally (such as *APOE* e4 and polygenic schizophrenia risk; though note that these were both weak associations that did not survive multiple comparisons correction) are, if causal, only likely to operate in old age, since they had not had any appreciable associations with brain structure preceding the follow-up waves of our study. Variables such as education and prior intelligence, which were related cross-sectionally but not longitudinally to brain structure, might represent correlates of developmental changes in brain structure from earlier in life, but appear not to be prospectively coupled with ageing-related changes in the indices of brain structure examined here.

Physical fitness was significantly related to healthier brain ageing in three of the five measures included here, and two of these relations (to changes in normal-appearing white matter and in general mean diffusivity) survived both the multivariate model and the MMSE exclusion. It was also one of the few variables that had a significant correlation both with baseline levels and with changes in multiple brain measures. Such results are in line with the literature on the effects of physical fitness on brain (and cognitive) ageing, some of which comes from randomized experiments and can thus test causality (Ahlskog et al. 2011). One caveat should be noted: whereas the measures we used (grip strength, lung function, and walk speed) are excellent general indicators of physical fitness (Enright et al. 2003), they may—at least partially—reflect pre-existing differences in bodily integrity (Frederiksen et al. 2002). The results do not, therefore, mean that improving physical fitness in old age will necessarily improve brain health, although they do leave open that possibility. Nevertheless, whether or not the relations we found are causal, our results show that differences in physical fitness may be informative for the prediction of future brain ageing.

Sex differences were found for grey matter both at baseline and in terms of change, but they ran in the opposite direction: males had significantly larger grey matter volume at baseline (as expected on the basis of prior studies: see the meta-analysis by Ruigrok et al. 2014), but also showed significantly greater loss of grey matter across the subsequent 3 years than females. This may be due to the ‘law of initial value’ (that the degree of change is affected by the baseline level; in this case, that individuals with initially-higher levels of a trait have ‘more to lose’ subsequently), or could represent a sex-specific ageing process, affecting males disproportionately during this period of life.

The two genetic factors we studied, the *APOE* e4 allele and polygenic risk for schizophrenia, showed small-sized relations to neurostructural decline. *APOE* e4 was linked to significantly faster decline in white matter volume and significantly faster accumulation of white matter hyperintensities (though this association did not survive multiple comparisons correction). As noted above, there has previously been mixed evidence for any detectable influence of *APOE* e4 on brain decline in healthy individuals (Josephs et al. 2008; Wishart et al. 2006), despite its being a well-replicated risk factor for Alzheimer’s-related brain pathology (Liu et al. 2015). It should be noted that the effects of *APOE* e4 were smaller in the analysis excluding individuals with low MMSE scores, implying that any *APOE* effects are stronger in individuals whose MMSE scores might indicate prodromal dementia (though the significant effects were small in the full sample, so this may also reflect the lower power of the secondary analysis). To our knowledge, no studies have tested the association of polygenic schizophrenia risk with longitudinal brain change. Suggestive evidence from the present study hints that polygenic schizophrenia risk may be a useful predictor of subsequent decline in brain structure (the relation did not survive multiple comparisons correction); if replicated, the reasons for this—a shared genetic aetiology (Lopez et al. 2015), or influences of schizophrenia genes on lifestyle factors that are detrimental to brain maintenance—would be uncertain. The polygenic schizophrenia risk score was chosen on the basis of work showing genetic correlations between schizophrenia and cognitive abilities, including in older age (Hagenaars et al. 2016; there have also been previous cognitive results in this sample; McIntosh et al. 2013), but polygenic risk scores for other conditions, such as stroke, may also be predictive of neurostructural changes and should be a target for future work.

Rather than relying on conventional multiple regression methods, which have biases with respect to overfitting (particularly in the presence of high multicollinearity), we used penalized regression—designed to reduce overfit—in our simultaneous models. Future studies of ageing-related brain changes could use our multivariate findings as a basis for investigations to more directly determine the replicability of these results.

All of the structural brain parameters we measured have previously been related both to baseline levels of and changes in at least some key cognitive functions, such as memory, reasoning, and processing speed (e.g. Charlton et al. 2010; Lövdén et al. 2014; Ritchie et al. 2015a, b). Future work should use more complex structural equation models to simultaneously investigate predictors of brain change alongside predictors of cognitive change: for instance, researchers could test whether fitness-related white matter changes mediate simultaneous changes in cognitive ability. Such an analysis, which is beyond the scope of the present paper, would take a step closer to a causal understanding of the antecedents and brain mechanisms of ageing-related cognitive decline (see Köhncke et al. 2016 for an example of this design with engagement in leisure activities as the predictor).

The present study is, to our knowledge, the largest-scale investigation of predictors of differential brain ageing to date. Compared to previous longitudinal studies of this nature (e.g. Enzinger et al. 2005), our sample was particularly large, enough to reduce (though not eliminate) the impact of scanner measurement noise on our estimates. We also assessed a large number of of predictors and structural indices, adding to our study’s comprehensiveness. Moreover, the narrow age range meant that the confounding effects of age were substantially reduced, and the cultural and ethnic homogeneity of the cohort limited other confounding effects. Where appropriate, we capitalized on latent variable modeling, which reduced the dimensionality of our predictors and eliminated measurement error (for instance, the use of general factors of FA and MD removed the influence of tract-specific measurement error), implementing latent difference score models of change over time.

The study has some limitations. First, our study period only lasted 3 years: such a brief period may not have resulted in substantial enough changes for us to reliably detect predictors with smaller effect sizes, even with our relatively large sample size (it is likely that the majority of the effects of these predictors are small); two waves of measurement also prevented us from examining any non-linearities in brain change. Second, healthier individuals are more likely to enroll in studies of this nature, and are less likely to drop out as the study continues. We implemented maximum likelihood estimation methods that reduce missing data bias resulting from longitudinal attrition, under the assumption that patterns of missingness in our outcomes that systematically relate to the unobserved scores on those outcomes are accounted for by non-missing scores on the variables included in our model. Nevertheless, restriction of range in baseline levels of our variables meant that we likely underestimated many effect sizes reported here. We might, for instance, have missed particularly debilitating brain change effects in individuals with very high baseline levels of allostatic load.

Third, the brain variables we used were relatively broad-brush: they indexed change in global grey and white matter, and in general white matter microstructure, but further research with finer-grained brain measures—examining particular grey matter regions or specific white matter tracts, which may themselves be more strongly linked to certain cognitive abilities—is required to test hypotheses relating potential predictors to specific brain areas (Maass et al. 2015). Fourth, our predictors were all time-invariant: we focused on the predictive value of measurements made at age 73 for declines in neural health between 73 and 76. However, a number of the parameters—such as physical fitness, allostatic load, and alcohol consumption—also change over time. Future studies should investigate whether changes in these parameters are coupled with changes in any of the brain markers assessed here. Additional waves of follow-up in the LBC1936 should allow us greater power to detect correlations with time-varying covariates (Rast and Hofer 2014).

This study investigated predictors of differential brain ageing between age 73 and 76 years. There was little correspondence between variables that were cross-sectionally associated with brain variables and those that predicted their changes, though physical fitness—among the strongest predictors in our analyses—was an exception. These results serve as a benchmark for future studies that attempt to replicate and validate the contributions of predictors of brain maintenance (Nyberg et al. 2012), and to estimate the extent to which their proximal effects on different brain structures mediate their ultimate effects on cognitive and functional decline.

## Electronic supplementary material

Below is the link to the electronic supplementary material.

Supplementary material 1 (DOCX 32 KB)
