# Supplementary material for: Risk and protective factors for structural brain ageing in the eighth decade of life
Source: Brain Struct Funct. 2017 Apr 19;222(8):3477–90. doi: 10.1007/s00429-017-1414-2 (PMC5676817; doi:10.1007/s00429-017-1414-2)
Supplement: Supplementary file 1 — Supplementary material 1 (DOCX 32 KB) [file 429_2017_1414_MOESM1_ESM.docx]

**Supplementary Material for:**

**Risk and protective factors for structural brain aging in the eighth decade of life**

Stuart J. Ritchie*, Elliot M. Tucker-Drob, Simon R. Cox, David Alexander Dickie, Maria del C. Valdés Hernández, Janie Corley, Natalie A. Royle, Paul Redmond, Susana Muñoz Maniega, Alison Pattie, Benjamin S. Aribisala, Adele M. Taylor, Toni-Kim Clarke, Alan J. Gow, John M. Starr, Mark E. Bastin, Joanna M. Wardlaw, Ian J. Deary

***Address correspondence to:** Dr. Stuart J. Ritchie, Centre for Cognitive Ageing and Cognitive Epidemiology, The University of Edinburgh, Edinburgh, EH8 9JZ, UK. Tel: +44 (0) 131 650 3453; Fax: +44 (0) 131 651 3190; Email: [stuart.ritchie@ed.ac.uk](mailto:stuart.ritchie@ed.ac.uk).

**Contents:**

Table S1, Table S2

*Table S1.* Associations between predictors and baseline brain measures at age 73 (figures correspond to the left heatmap shown in Figure 4 of the main document). *p*_adj_ = *p*-values after False Discovery Rate correction. *p*- and *p*_adj_-values < .05 shown in boldface.

| Brain measure | Predictor | Standardized *β* | SE | *p* | *p*_adj_ |
| --- | --- | --- | --- | --- | --- |
| Grey matter volume | Sex (male) | .576 | .026 | **9.58×10^-109^** | **5.27×10^-107^** |
|  | Physical fitness | .218 | .050 | **1.30×10^-05^** | **7.95×10^-05^** |
|  | Allostatic load | -.229 | .037 | **6.05×10^-10^** | **5.60×10^-09^** |
|  | Health cond. | -.111 | .032 | **5.23×10^-04^** | **.002** |
|  | SES | .233 | .042 | **2.90×10^-08^** | **2.28×10^-07^** |
|  | IQ | .201 | .030 | **2.08×10^-11^** | **2.87×10^-10^** |
|  | Education | .087 | .032 | **.007** | **.019** |
|  | Smoking | -.074 | .032 | **.021** | .052 |
|  | Alcohol | .038 | .036 | .291 | .415 |
|  | *APOE* e4 | .045 | .033 | .173 | .291 |
|  | Scz. PGR | .027 | .035 | .440 | .563 |
| White matter volume | Sex (male) | .510 | .029 | **3.14×10^-69^** | **8.64×10^-68^** |
|  | Physical fitness | .164 | .055 | **.003** | **.010** |
|  | Allostatic load | -.134 | .040 | **8.08×10^-04^** | **.003** |
|  | Health cond. | -.097 | .034 | **.004** | **.014** |
|  | SES | .304 | .043 | **1.55×10^-12^** | **2.85×10^-11^** |
|  | IQ | .198 | .032 | **6.11×10^-10^** | **5.60×10^-09^** |
|  | Education | .126 | .033 | **1.34×10^-04^** | **5.69×10^-04^** |
|  | Smoking | -.069 | .033 | **.037** | .080 |
|  | Alcohol | -.001 | .038 | .979 | .979 |
|  | *APOE* e4 | .056 | .034 | .100 | .196 |
|  | Scz. PGR | .058 | .036 | .107 | .203 |
| White matter hyperintensity volume | Sex (male) | -.009 | .039 | .817 | .883 |
|  | Physical fitness | .250 | .061 | **4.16×10^-05^** | **2.08×10^-04^** |
|  | Allostatic load | -.022 | .047 | .640 | .733 |
|  | Health cond. | -.091 | .039 | **.020** | .051 |
|  | SES | .146 | .053 | **.006** | **.018** |
|  | IQ | .049 | .040 | .221 | .357 |
|  | Education | -.016 | .039 | .682 | .765 |
|  | Smoking | -.065 | .039 | .096 | .195 |
|  | Alcohol | -.025 | .044 | .570 | .683 |
|  | *APOE* e4 | -.042 | .040 | .294 | .415 |
|  | Scz. PGR | .009 | .043 | .834 | .883 |
| General fractional anisotropy | Sex (male) | -.005 | .042 | .905 | .939 |
|  | Physical fitness | .324 | .064 | **4.14×10^-07^** | **2.84×10^-06^** |
|  | Allostatic load | -.205 | .049 | **2.87×10^-05^** | **1.58×10^-04^** |
|  | Health cond. | -.106 | .042 | **.012** | **.032** |
|  | SES | .126 | .062 | **.042** | .089 |
|  | IQ | .092 | .043 | **.032** | .077 |
|  | Education | -.023 | .042 | .584 | .683 |
|  | Smoking | -.089 | .042 | **.034** | .078 |
|  | Alcohol | .010 | .048 | .835 | .883 |
|  | *APOE* e4 | .024 | .043 | .577 | .683 |
|  | Scz. PGR | .043 | .046 | .350 | .469 |
| General mean diffusivity | Sex (male) | .037 | .042 | .378 | .495 |
|  | Physical fitness | .386 | .098 | **8.19×10^-05^** | **3.75×10^-04^** |
|  | Allostatic load | -.051 | .051 | .317 | .436 |
|  | Health cond. | -.057 | .042 | .175 | .291 |
|  | SES | .064 | .061 | .294 | .415 |
|  | IQ | .004 | .060 | .947 | .964 |
|  | Education | .049 | .041 | .232 | .365 |
|  | Smoking | -.098 | .066 | .138 | .252 |
|  | Alcohol | .052 | .047 | .269 | .410 |
|  | *APOE* e4 | -.058 | .042 | .167 | .291 |
|  | Scz. PGR | .030 | .045 | .505 | .631 |

*Note:* Health cond. = number of health conditions; SES = Socioeconomic status; IQ = prior intelligence; Scz. PGR = Polygenic risk score for schizophrenia. *p*_adj_ = *p*-values after False Discovery Rate correction. *p*- and *p*_adj_-values < .05 shown in boldface.

*Table S2.* Associations between predictors and change in brain measures from age 73 to 76 (figures correspond to right heatmap shown in Figure 4 of the main document).

| Brain measure | Predictor | Standardized *β* | SE | *p* | *p*_adj_ |
| --- | --- | --- | --- | --- | --- |
| Grey matter volume | Sex (male) | -.218 | .044 | **7.25×10^-07^** | **3.99×10^-05^** |
|  | Physical fitness | -.077 | .082 | .348 | .653 |
|  | Allostatic load | .094 | .054 | .082 | .321 |
|  | Health cond. | -.043 | .045 | .339 | .653 |
|  | SES | .071 | .063 | .260 | .653 |
|  | IQ | .030 | .047 | .523 | .730 |
|  | Education | .011 | .044 | .803 | .901 |
|  | Smoking | .046 | .047 | .328 | .653 |
|  | Alcohol | -.021 | .052 | .686 | .858 |
|  | *APOE* e4 | -.022 | .046 | .632 | .809 |
|  | Scz. PGR | -.033 | .050 | .509 | .730 |
| White matter volume | Sex (male) | .086 | .046 | .062 | .288 |
|  | Physical fitness | .183 | .082 | **.026** | .176 |
|  | Allostatic load | .0001 | .055 | .999 | 1.000 |
|  | Health cond. | -.002 | .046 | .965 | 1.000 |
|  | SES | .132 | .064 | **.039** | .215 |
|  | IQ | .081 | .048 | .092 | .336 |
|  | Education | .050 | .045 | .267 | .653 |
|  | Smoking | -.033 | .048 | .492 | .730 |
|  | Alcohol | .029 | .053 | .584 | .765 |
|  | *APOE* e4 | -.098 | .046 | **.033** | .202 |
|  | Scz. PGR | -.127 | .050 | **.011** | .122 |
| White matter hyperintensity volume | Sex (male) | -.025 | .043 | .561 | .753 |
|  | Physical fitness | .135 | .074 | .068 | .288 |
|  | Allostatic load | .044 | .052 | .397 | .666 |
|  | Health cond. | -.069 | .043 | .109 | .373 |
|  | SES | .054 | .060 | .368 | .653 |
|  | IQ | -.008 | .044 | .856 | .923 |
|  | Education | .050 | .045 | .267 | .653 |
|  | Smoking | .033 | .048 | .492 | .730 |
|  | Alcohol | -.115 | .049 | **.019** | .174 |
|  | *APOE* e4 | -.124 | .043 | **.004** | .054 |
|  | Scz. PGR | -.043 | .047 | .360 | .653 |
| General fractional anisotropy | Sex (male) | .061 | .059 | .301 | .653 |
|  | Physical fitness | -.035 | .105 | .739 | .883 |
|  | Allostatic load | -.025 | .070 | .721 | .881 |
|  | Health cond. | -.040 | .060 | .505 | .730 |
|  | SES | -.085 | .088 | .334 | .653 |
|  | IQ | -.039 | .061 | .523 | .730 |
|  | Education | -.015 | .058 | .796 | .901 |
|  | Smoking | -.015 | .060 | .803 | .901 |
|  | Alcohol | -.099 | .067 | .140 | .451 |
|  | *APOE* e4 | .000 | .060 | 1.000 | 1.000 |
|  | Scz. PGR | -.074 | .064 | .248 | .653 |
| General mean diffusivity | Sex (male) | -.053 | .058 | .361 | .653 |
|  | Physical fitness | .386 | .098 | **8.19×10^-05^** | **.002** |
|  | Allostatic load | -.154 | .068 | **.024** | .176 |
|  | Health cond. | -.170 | .057 | **.003** | .052 |
|  | SES | .020 | .090 | .824 | .907 |
|  | IQ | .057 | .060 | .342 | .653 |
|  | Education | .048 | .057 | .400 | .666 |
|  | Smoking | -.087 | .066 | .187 | .573 |
|  | Alcohol | -.042 | .067 | .531 | .730 |
|  | *APOE* e4 | -.008 | .058 | .890 | .942 |
|  | Scz. PGR | -.115 | .063 | .068 | .288 |

*Note:* Health cond. = number of health conditions; SES = Socioeconomic status; IQ = prior intelligence; Scz. PGR = Polygenic risk score for schizophrenia. *p*_adj_ = *p*-values after False Discovery Rate correction. *p*- and *p*_adj_-values < .05 shown in boldface.
